# Latrotoxin-Induced Neuromuscular Junction Degeneration Reveals Urocortin 2 as a Critical Contributor to Motor Axon Terminal Regeneration

**DOI:** 10.3390/ijms23031186

**Published:** 2022-01-21

**Authors:** Giorgia D’Este, Marco Stazi, Samuele Negro, Aram Megighian, Florigio Lista, Ornella Rossetto, Cesare Montecucco, Michela Rigoni, Marco Pirazzini

**Affiliations:** 1 Department of Biomedical Sciences, University of Padova, Via Ugo Bassi 58/B, 35131 Padova, Italy; giorgia.deste@studenti.unipd.it (G.D.); marco.stazi@phd.unipd.it (M.S.); samuele.negro@aopd.veneto.it (S.N.); aram.megighian@unipd.it (A.M.); ornella.rossetto@unipd.it (O.R.); cesare.montecucco@gmail.com (C.M.); 2 U.O.C. Clinica Neurologica, Azienda Ospedale-Università Padova, Via Giustiniani 5, 35128 Padova, Italy; 3 Padova Neuroscience Center, University of Padova, Via Giuseppe Orus 2, 35131 Padova, Italy; 4 Army Medical Center, Scientific Department, Via Santo Stefano Rotondo 4, 00184 Roma, Italy; florigio.lista@esercito.difesa.it; 5 Italian Research Council, Institute of Neuroscience, University of Padova, Via Ugo Bassi 58/B, 35131 Padova, Italy; 6 CIR-Myo, Department of Biomedical Sciences, University of Padova, Via Ugo Bassi 58/B, 35131 Padova, Italy

**Keywords:** α-Latrotoxin, neuromuscular junction, nerve degeneration, nerve regeneration, Urocortin 2, CRHR2 receptor

## Abstract

We used α-Latrotoxin (α-LTx), the main neurotoxic component of the black widow spider venom, which causes degeneration of the neuromuscular junction (NMJ) followed by a rapid and complete regeneration, as a molecular tool to identify by RNA transcriptomics factors contributing to the structural and functional recovery of the NMJ. We found that Urocortin 2 (UCN2), a neuropeptide involved in the stress response, is rapidly expressed at the NMJ after acute damage and that inhibition of CRHR2, the specific receptor of UCN2, delays neuromuscular transmission rescue. Experiments in neuronal cultures show that CRHR2 localises at the axonal tips of growing spinal motor neurons and that its expression inversely correlates with synaptic maturation. Moreover, exogenous UCN2 enhances the growth of axonal sprouts in cultured neurons in a CRHR2-dependent manner, pointing to a role of the UCN2-CRHR2 axis in the regulation of axonal growth and synaptogenesis. Consistently, exogenous administration of UCN2 strongly accelerates the regrowth of motor axon terminals degenerated by α-LTx, thereby contributing to the functional recovery of neuromuscular transmission after damage. Taken together, our results posit a novel role for UCN2 and CRHR2 as a signalling axis involved in NMJ regeneration.

## 1. Introduction

The neuromuscular system of vertebrates is composed of spinal motor neurons and skeletal muscles that together control life-essential tasks like locomotion and breathing. A key anatomical structure of this system is the neuromuscular junction (NMJ), the specialised synapse between the motor neuron axon terminal (MAT) and the muscle fibre (MF). The NMJ converts the electric stimuli running along motor axons into acetylcholine (Ach) release that is sensed by specific receptors located in the MF to elicit muscle contraction [1,2]. A third essential NMJ component is a carpet of perisynaptic Schwann cells (PSC) [3], glial cells of the peripheral nervous system (PNS) that cover the MAT and oversee NMJ function and maintenance [4].

Due to the lack of anatomical barriers, the NMJ is exposed and freely permeable to toxins of any kind and has become throughout evolution a target of bacteria, animals, and plants to prey on and defend against opponent species [5,6,7]. In addition, a variety of different types of damage, including mechanical traumas as well as autoimmune attacks, can affect peripheral nerve terminals and the NMJ, leading to nerve degeneration and neuromuscular impairment [1,2,8,9,10]. At the same time, peripheral axons and the NMJ have maintained and refined throughout evolution the remarkable ability to self-repair and even regenerate after damage, most likely because the NMJ must remain functional throughout life to secure neuromuscular transmission, which is essential for survival [2].

NMJ maintenance is accomplished by a fine-tuning of signals exchanged among MAT, PSC and MF, which also takes place after MAT damage to support its structural regeneration and functional recovery [2,8,11]. This process is orchestrated by a core set of proteins and signalling pathways identified throughout years of intense research, although the complete picture of the molecular determinants of such intercellular cross talk remains largely unknown [12].

To expand the current knowledge of the process of peripheral neuroregeneration, and to detangle the complex signalling occurring among MAT, PSC and MF, we recently devised an innovative method based on the use of α-latrotoxin (α-LTx), the main neurotoxic component of the black widow spider venom. α-LTx induces a selective degeneration of the MAT followed by a rapid structural and functional regeneration, with little if any inflammation [10]. A sublethal amount of α-LTx was locally injected in the mouse hind limb, where it led to a controlled degeneration of the MAT and to its rapid regeneration in a few days [10,13,14]. Then, isolation of the NMJs and sequencing of their RNA provided a time-resolved mRNA profiling of the NMJ during the entire process of degeneration and regeneration [15]. By this approach, we found that the molecular axis composed by the chemokine CXCL12α and its receptor CXCR4 acts as a potent driver of MAT regeneration [15], and that the small molecule compound NUCC-390, an agonist of CXCR4 [16], is a strong promoter of nerve regeneration in several models of PNS damage, spanning from neurotoxic venoms to traumas [17,18,19].

In this work, we re-investigated the transcriptomic profile of the NMJ at the different stages of intoxication by α-LTx and report the unexpected finding that the small stress hormone Urocortin-2 (UCN2) contributes to MAT regeneration. We found that *UCN2* mRNA is strongly induced at injured NMJs of two different mouse muscles, and that its action via CRHR2 contributes to structural regeneration and functional recovery of the NMJ. UCN2 boosts nerve growth in cultured neurons by engaging its specific receptor CRHR2, which is consistently expressed at the tips of growing axons of cultured spinal cord motor neurons (SCMN).

## 2. Results

### 2.1. UCN2 mRNA Is Strongly Up-Regulated after NMJ Degeneration Induced by α-LTx

**Figure 1:**
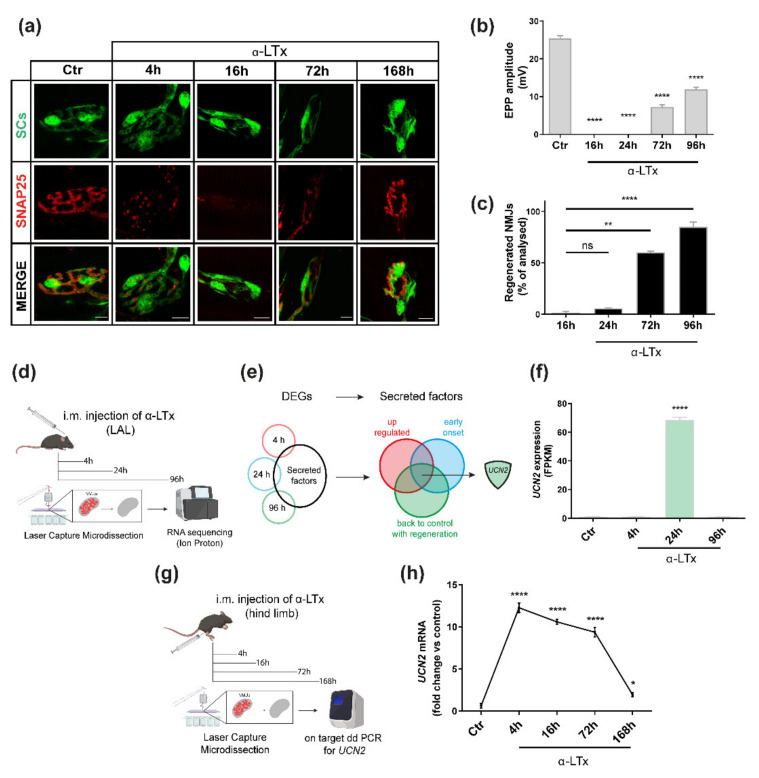
Time course of NMJ degeneration and regeneration by α-LTx reveals a strong upregulation of the *UCN2* gene. (**a**) Time course of MAT degeneration and regeneration induced by α-LTx in soleus muscles assessed by immunostaining at 4, 16, 72, 168 h after intoxication. SCs (green) are GFP-positive, SNAP25 (red) identifies the presynaptic nerve terminals. Scale bars = 10 μm. (**b**) Time course of loss and progressive recovery of motor axon activity by evoked EPP recordings in soleus muscles at the indicated time points. Histograms show the average amplitude of evoked EPPs ± SD. *n* = 4 animals, 11 fibres each. **** *p* < 0.0001. ANOVA followed by post hoc Tukey test. (**c**) Quantification of regenerated NMJs in soleus muscles at the indicated time points after α-LTx injection. Regenerated NMJs display both pre- and post-synaptic markers (VAMP-1 and nAChR stained with α-BTx, respectively). Each bar represents the mean ± SD percentage of recovered NMJs compared to non-treated animals. **** *p* < 0.0001, ** *p* < 0.01 by ANOVA followed by post hoc Tukey test. (**d**) Experimental pipeline of NMJ transcriptome profiling throughout the process of degeneration and regeneration caused by α-LTx. Mice were injected with the toxin close to LAL muscle, which was then isolated and snap-frozen at the indicated time points. Single NMJs were isolated by LCM from cryo-sliced muscles. At least 150 NMJs were pooled and their RNA extracted, amplified and sequenced with the Ion Torrent technology. (**e**) Scheme depicting the workflow and criteria used to process the NMJ dataset. (**f**) Graph showing *UCN2* mRNA profile, expressed as FPKM (fragments per kilobase of exon per million fragments mapped) during MAT degeneration and regeneration induced by α-LTx in LAL muscles. Data are presented as mean ± SD. **** *p* < 0.0001 (ctr vs. 24 h) from 3 independent replicates. (**g**) Experimental workflow to assess *UCN2* mRNA levels in soleus muscles 4, 16, 72, 168 h after local injection of α-LTx. Time points were chosen on the basis of the time course of MAT degeneration and regeneration by α-LTx as described above. (**h**) *UCN2* mRNA levels measured via digital PCR on NMJs isolated by LCM from soleus muscles. Data are expressed as fold change compared to control. Each bar represents mean ± SD. *n* = 4 animals. **** *p* < 0.0001, * *p* < 0.05. ANOVA followed by *post hoc* Tukey test.

α-LTx is a pore forming toxin that selectively binds to and generates holes in the presynaptic membrane that convey massive Ca^++^ entry into axon terminals leading to its rapid, synchronous and complete degeneration, with ensuing neuroparalysis [7,20]. When injected in sublethal amounts into a muscle, α-LTx causes the local degeneration of the MAT and neuromuscular paralysis, which; however, are quickly followed by the complete regeneration and recovery of NMJ activity [10]. In mice, this process takes a few days, and can be assessed by microscopy using specific presynaptic markers like SNAP-25 (Figure 1a), and by electrophysiology via monitoring the evoked endplate potentials (EPPs) (Figure 1b). Four hours after α-LTx injection, the MAT is destroyed, and neurotransmission blunted. MAT debris are engulfed by PSC, which clear NMJ area and initiate the regeneration process [13]. At 24 h, the MAT is reduced to a stump that progressively regrows, as indicated by the gradual re-appearance of SNAP-25 and neurotransmission recovery. At 96 h, MAT regeneration is almost complete and NMJ recovery of function is halfway (Figure 1b,c). Within one week (i.e., 168 h), regenerated NMJs look and perform as they did before damage.

From an experimental point of view, such a rapid time course is very convenient, and qualifies α-LTx as a valuable tool to identify factors involved in neuroregeneration. Therefore, we isolated by laser capture microdissection (LCM) the NMJs at different time points after α-LTx injection and then performed a transcriptional analysis to examine how gene expression changes throughout degeneration and regeneration (Figure 1d). We used the *Levator auris longus* (LAL), a thin and flat muscle amenable to LCM and imaging, and compared the transcriptomes of control NMJs with those collected 4, 24 and 96 h after α-LTx injection [15]. To restrict our analysis to transcripts encoding for potential neuro-regenerative factors, we screened the list of differentially expressed genes (DEGs) according to the workflow shown in Figure 1e. As a first filter, we considered the DEGs present in at least one of the 3 time points and endowed with a secretion signal or known to be released, e.g., growth factors, cytokines, chemokines, hormones, and small peptides. Among those, we further restricted the analysis to those transcripts that were (i) upregulated, as factors fostering nerve re-growth are expected to be induced after damage, (ii) early activated after degeneration, as pro-regenerative signals are known to be expressed soon after degeneration, and (iii) down-regulated back to control levels once regeneration has been attained, as these signals are expected to be shut down after recovery.

We focused on the *UCN2* transcript, which displays a 60-fold higher peak of expression at 24 h (a time point when the NMJ area has been cleared from debris and regeneration is ready to start), and then decreases back to controls (Figure 1f). Of note, a similar pattern of expression of the *UCN2* transcript is observed also in soleus muscles upon α-LTx injection in the mouse hind limb (Figure 1g), as assessed by digital PCR (Figure 1h), suggesting that the increase in *UCN2* mRNA expression at the NMJ is a general response to nerve terminal degeneration. Notably, *UCN2* upregulation in the soleus is even anticipated and maintained longer throughout MAT re-growth, fading away when regeneration is attained.

### 2.2. UCN2 Participates in NMJ Regeneration via CRHR2

The *UCN2* gene encodes for urocortin 2 (UCN2), a 38-aminoacid neuropeptide belonging to the corticotropin-releasing hormones (CRH) family, originally described as critical regulators of the hypothalamic-pituitary-adrenal axis during the stress response [21]. These peptides are mainly expressed in the brain but also in peripheral tissues and glands, where they modulate energy and cardiovascular homeostasis, reproduction behaviour, immune and inflammatory responses, and several endocrine functions through two specific GPCR receptor named CRHR1 and CRHR2 [22,23].

The peculiar expression profile of *UCN2* mRNA at the intoxicated NMJ prompted us to examine whether the encoded protein is involved in MAT regeneration. Previous work shows that muscle cells express UCN2 [24]. We thus hypothesised that myofibres could release UCN2 in response to denervation, which may act as a paracrine factor fostering MAT re-growth.

A convenient way to test this possibility is the use of an antibody able to sequester the peptide thus neutralising its biological action, as we did previously to show the pro-regenerative function of CXCL-12α released by PSC [15]. Unfortunately, we did not find any suitable antibody able to neutralise UCN2. Therefore, we used a specific antagonist of the CRHR2 receptor called Astressin 2B (A2B) that strongly inhibits the activity of CRHR2 [25].

**Figure 2:**
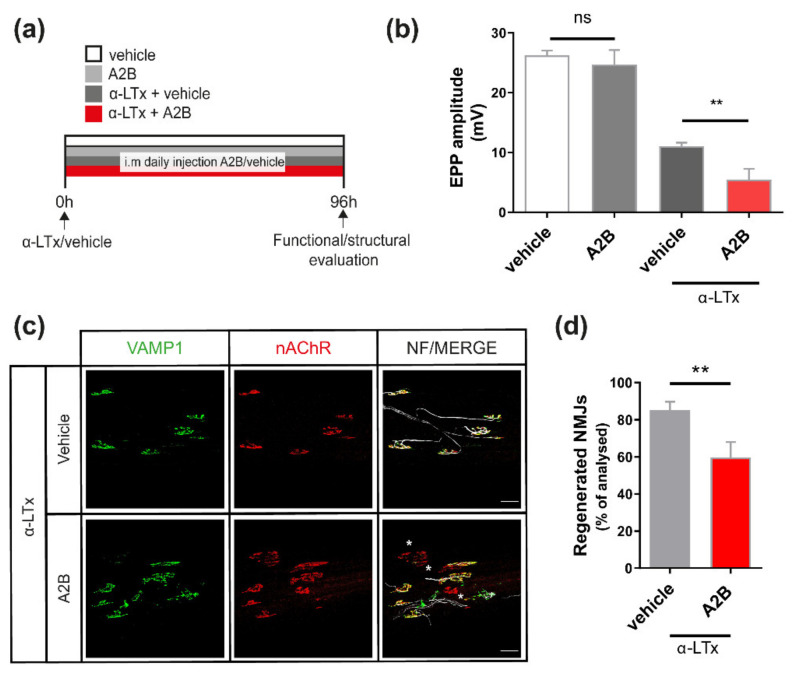
The CRHR2 receptor participates in the structural and functional recovery of the NMJ. (**a**) Scheme illustrating the treatment protocol, consisting of daily i.m. injections of either vehicle or the CRHR2 antagonist A2B up to 96 h after α-LTx injection, and the analysis performed to assess the functional and structural status of the NMJ. (**b**) Electrophysiological recordings of evoked EPPs from intoxicated soleus muscles after either vehicle of A2B administration. Each bar represents mean ± SD. *n* = 4 animals, 11 fibres. ** *p* < 0.01, ns *p* > 0.05. ANOVA followed by post hoc Tukey test. (**c**) Representative immunostaining and (**d**) quantification performed on soleus muscles after intoxication with α-LTx and treatment with either vehicle or A2B. Regenerated NMJs display both pre- and post-synaptic markers (VAMP-1 in green and nAChR in red, respectively). White asterisks indicate still degenerated NMJs (lack or little VAMP-1 signal). The right panel shows a magnification. Scale bars: 50 µm. Bars in (**d**) represent the mean± SD percentage of regenerated NMJs. ** *p* < 0.01 by Student’s *t*-test.

For this purpose, we injected α-LTx in the hind limb to induce nerve degeneration; then, we treated animals with i.m. injections of A2B (or vehicle) once a day for 96 h, and evaluated NMJ function via electrophysiology and imaging (Figure 2a). The dose used (200 μg/kg) was within the concentration range at which A2B has been reported to be effective in peripheral tissue explants [25]. The final time point (96 h) corresponds to 50% of NMJ functionality and allows an easier estimation of the effects of CRHR2 inhibition on neuromuscular recovery. Figure 2b shows that A2B treatment halved the average amplitude of the evoked EPPs, indicating a significant delay in the functional recovery of neurotransmission. At the same time, A2B treatment had a negligible effect on the evoked EPPs in control muscles, meaning that CRHR2 inhibition does not affect neurotransmission per se. Consistent with the delay in neurotransmission recovery, A2B treatment negatively affects the structural regeneration of the NMJs (Figure 2c), as assessed by counting the number of regenerated NMJs via imaging (Figure 2d), the latter being positive for VAMP-1 in the pre-synapse [26], a marker indicative of regeneration of the machinery responsible for neurotransmission.

### 2.3. CRHR2 Expression Is Associated with Axonal Growth

The UCN2 receptor CRHR2 is mainly expressed in the central nervous system (CNS), but also in peripheral tissues including the cardiovascular system, the gastrointestinal tract and skeletal muscles [23]. Importantly, CRHR2 expression in skeletal muscles is restricted to the neuronal compartment, including the MAT [27]. This led us to hypothesise that after MAT damage, denervated myofibres release UCN2 that, by acting on neuronal CRHR2, fosters MAT regeneration to re-establish the lost synaptic contacts.

**Figure 3:**
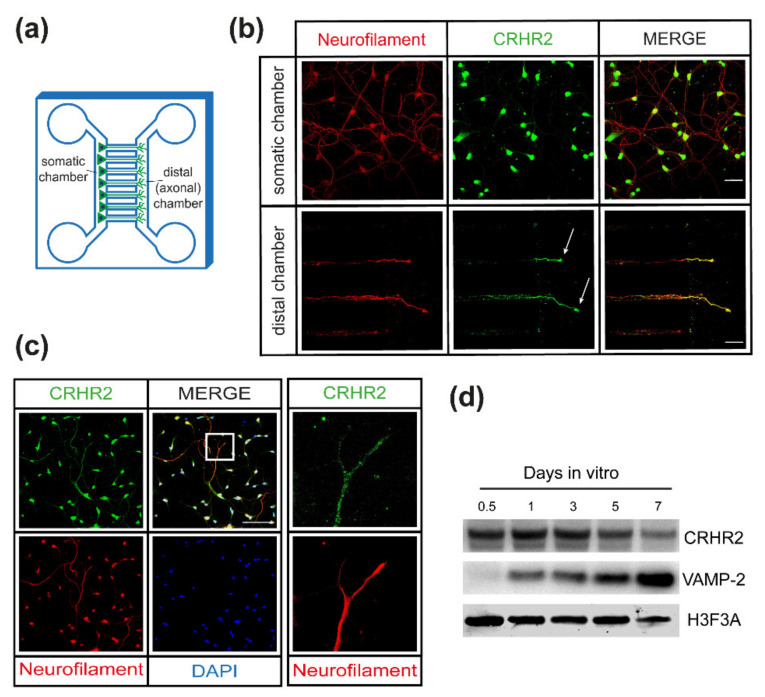
CRHR2 localises at the axonal tips of growing neurons and its expression decreases with synaptogenesis. (**a**) Graphical representation of the microfluidic devices (MFC) used in the study, showing the somatic chamber, where neurons are plated, connected to the distal (axonal) one by microgrooves where axons elongate in a directional manner. (**b**) Representative immunostaining of SCMNs cultured for 7 days in MFC showing the expression of neurofilaments (red, to label the axonal cytoskeleton) and CRHR2 (green) on axon tips (arrows). Top panels show the somatic chamber and bottom panels the axonal one. Scale bars = 10 μm. (**c**) Representative immunostaining of SCMNs after 24 h of culture at low density showing the expression of neurofilaments (red) and CRHR2 (green). The right panel shows a magnification of the inset. Scale bar = 10 μm. (**d**) Representative Western blot showing the expression of CRHR2 and VAMP-2 in CGNs at different days in culture. Expression of histone H3 represents a loading control.

To test this possibility, we devised a series of in vitro experiments. First, we monitored CRHR2 expression in primary spinal cord motor neurons (SCMNs) cultured in microfluidic devices. In this system, neurons are plated in a somatic chamber that is separated from a “nerve terminal” one by microsized grooves corresponding to the axonal compartment (Figure 3a). By immunostaining, we found expression of CRHR2 in the distal chamber, in axonal tips at the level of axonal growth cones (Figure 3b). Conversely, in the somatic chamber, where axons not entering the microsized grooves are free to form synaptic contacts with other axons and dendrites, CRHR2 signal was mainly found within the cell body with little, if any, expression in axons and synaptic contacts.

Second, we seeded SCMNs on standard coverslips at low density and examined the distribution of CRHR2. We found the receptor highly expressed along the whole axons and axonal tips, and only to a lesser extent in the few inter-neuronal contacts already formed (Figure 3c).

Third, we monitored CRHR2 expression throughout the maturation steps of the neuronal culture, and compared it with that of VAMP-2, a marker of neuronal differentiation and synapse formation. In this case, we used a culture of rat cerebellar granular neurons (CGNs) because of their higher purity with respect to rat SCMNs [28]. As shown in Figure 3d, CRHR2 displays a very strong signal right after plating, which then progressively disappears, at variance from VAMP-2 level that increases with time, suggesting that CRHR2 expression progressively declines as synaptogenesis progresses.

### 2.4. UCN2 Boosts Axon Growth in Cultured SCMNs via CRHR2 and Fosters NMJ Regeneration

**Figure 4:**
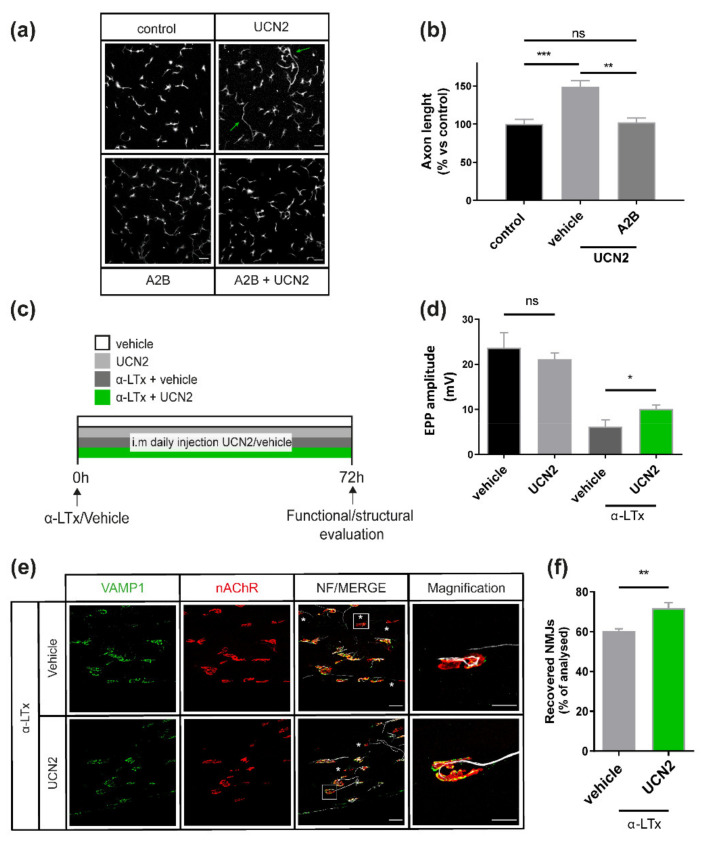
The UCN2-CRHR2 axis promotes axonal growth of cultured SCMNs and NMJ regeneration after acute damage. (**a**) Representative images of neurofilament staining used as reporter for axon length in SCMNs cultured for 24 h either in normal culture medium (NC) or NC supplemented with UCN2, A2B or their combination. (**b**) Quantification of the average axonal length after the indicated treatments. Three independent experiments were performed, and at least 100 neurons per condition measured. Bars represent mean ± SD. Statical analysis was performed with ANOVA followed by *post hoc* Tukey test; *** *p* < 0.001, ** *p* < 0.01, ns *p* > 0.05. (**c**) Scheme illustrating the treatment protocol, consisting of daily i.m. injections of either vehicle or recombinant UCN2 for 72 h after α-LTx treatment, followed by functional and structural assessment of NMJ status. (**d**) Electrophysiological assessment of NMJ function by evoked EPPs recordings following either vehicle or UCN2 administration. Each bar represents mean ± SD. *n* = 4 animals, 11 fibres. * *p* < 0.05, ns *p* > 0.05. ANOVA followed by post hoc Tukey test. (**e**) Representative immunostaining and (**f**) quantification performed on soleus muscles after intoxication and treatment with either vehicle or UCN2. Regenerated NMJs are positive for both pre- and post-synaptic markers (VAMP-1 in green and nAChR in red, respectively). White asterisks indicate still degenerated NMJs (absent or faint VAMP1 signal). Right panels are magnifications of the indicated NMJs. Scale bars: 50 µm, 20 µm for magnification. Each bar in (**f**) represents the mean ± SD percentage of recovered NMJs. ** *p* < 0.01 by Student’s *t*-test.

The above-described results suggest that the molecular axis CRHR2-UCN2 promotes axon growth. To test this hypothesis, we supplemented the neuronal culture medium with recombinant UCN2 right after SCMN plating, and evaluated the rate of axon growth via imaging of neurofilaments (NF), a major component of the axonal cytoskeleton (Figure 4a). We report that UCN2 administration leads to 50% increase in the length of axons grown in the first 24 h. Importantly, this effect is CRHR2-mediated, as it is prevented by the antagonist A2B (Figure 4b).

As NMJ regeneration recapitulates several aspects of neuronal development and axon growth [12,29], and CRHR2 inhibition delays MAT re-growth (Figure 2), we tested whether UCN2 enhances the re-growth of the MAT after damage. We injected α-LTx in the hind limb, treated mice with i.m. injections of UCN2 (or vehicle) once a day for 72 h, and then evaluated NMJ morphology and function as before (Figure 4c). In this case, we stopped the experiment at 72 h, a time point at which structural and functional recovery of the MAT is still limited, thus making easier to evaluate the effects of UCN2 as pro-regenerative factor. While UCN2 treatment did not affect neurotransmission of control muscles, it significantly speeded up the functional recovery after α-Ltx treatment, as indicated by the larger average amplitude of evoked EPPs (Figure 4d). Consistently, UCN2 treatment led to a faster regrowth of degenerated NMJs (Figure 4e,f).

## 3. Discussion

Spiders, snakes, scorpions, ticks, and other animals have refined throughout evolution complex venomous mixtures of toxins and bioactive peptides with the ultimate scope to prey or defend in the biological arena of wildlife. An incredible number of biomolecules has evolved to selectively hit specific physiological functions and/or cellular targets that are essential for survival. Decades of scientific efforts have demonstrated the importance of studying venoms, toxins, and natural biomolecules to dissect their pathogenic action on one hand, and the cell target physiology on the other. In addition, the same toxins and bioactive molecules have revealed therapeutical properties or proved to be sophisticated tools to study the complexity of biological systems [5,6,30,31,32].

We provide here a relevant example that a toxin can be a tool to induce a controlled and highly reproducible degeneration of the MAT and study the ensuing peripheral regeneration, a highly conserved process of vertebrates, including humans [10]. One additional advantage of our model is that α-LTx causes a localised neuronal damage without inflammation, thus facilitating the identification of signals involved in the process [10,12]. We report here an unprecedented role for UCN2 as an important contributor of NMJ regeneration. This conclusion is supported by several findings: (i) UCN2 mRNA is upregulated in skeletal muscles after MAT damage, (ii) inhibition of CRHR2, the specific membrane receptor of UCN2, delays MAT regeneration and functional recovery, (iii) UCN2 enhances axonal growth in cultured MNs via CRHR2, expressed at axon tips, and (iv) exogenous UCN2 fosters the structural regeneration of the NMJ, thus accelerating neurotransmission rescue.

Urocortins and CRH neuropeptides are abundantly expressed in different brain regions of the CNS and are mainly known for their role in the regulation of stress responses via the hypothalamic-pituitary-adrenal axis [21,33]. Beyond the hormonal activity, previous reports revealed a role for UCN2 and other CRH family members in synaptic formation and in the shaping of novel synaptic circuits by eliciting different intra-neuronal signalling pathways via specific CRHR receptors [34,35,36].

Our results indicate that UCN2 exerts a similar effect at the NMJ by stimulating the CRHR2 receptor, thus providing an important contribution in the regeneration of the neuromuscular synapse after an acute degeneration. This is in line with previous results showing UCN2 expression by myofibres and that of CRHR2 by the MAT [24,27]. Moreover, our findings are in accordance with the experimental evidence that the *UCN* gene is induced in retinal ganglion cells after optic nerve damage, and that its overexpression promotes both neuron resilience to death after damage, and axonal regeneration and regrowth [37]. Consistently, we report here that UCN2 promotes axonal elongation during in vitro MN development, and that this effect is mediated by CRHR2, whose expression fades away with the progressive formation of synaptic contacts. Overall, these results extend previous findings reporting that the CRH/UCN1-CRHR1 axis mediates neurite sprouting, outgrowth and elongation in hippocampal-like cells [38] and increases dendritic elongation and branching in central neurons [39]. Considering that MAT regeneration recapitulates several aspects of MN and NMJ development [12], these results, together with the finding that the UCN2-CRHR2 axis promotes MAT regeneration, appoint the CRHR2-UCN2 axis as a potential neurodevelopmental pathway working at the crossroad between MAT elongation and NMJ formation. This possibility will be investigated in future studies.

Neurodegeneration by α-LTx is triggered by a presynaptic membrane damage leading to cytosolic and mitochondrial Ca^2+^ overload and ensuing dismantling of the MAT due to mitochondrial dysfunction and activation of Ca^2+^-dependent proteases [13,40,41,42]. This pathogenic mechanism is shared by other pathologies affecting the NMJ, including (i) autoimmune neurodegenerative disorders triggered by antibodies raised against presynaptic NMJ antigens, like the Guillain–Barrè and Miller–Fisher syndromes [43,44,45,46,47], and (ii) snake phospholipase A2 neurotoxins that cause a reversible degeneration of the MAT leading to a transient [48], yet potentially lethal neuroparalysis [7,10,49,50]. It is, therefore, conceivable that the pro-regenerative effects of the UCN2-CRHR2 couple reported in the α-LTx model can be extended also to these conditions. One cannot exclude that the UCN2-CRHR2 axis plays a role also in chronic neurodegenerative contexts (e.g., in amyotrophic lateral sclerosis, where distal instability due to NMJ dysfunction is emerging as a primary pathogenic mechanism). However, this possibility deserves an ad hoc investigation.

In conclusion, our results provide a double message: (i) animal toxins are potent tools to study cell and tissue physiology, and (ii) UCN2 could represent a lead compound for the development of novel therapeutics or pharmacological treatments to counteract NMJ degeneration and dysfunction.

## 4. Materials and Methods

### 4.1. Antibodies and Reagents

Unless otherwise stated, all other reagents were from Sigma Aldrich (St Louis, MO, USA). The following primary antibodies were employed at the indicated dilutions: anti-VAMP-1 (generated as described in [51], 1:200 IF), anti-VAMP-2 (Synaptic System, Göttingen, Germany, cat. 104211, 1:1000 Immunoblot), anti-CRHR2 (ThermoFisher Scientific, Waltham, MA, USA, cat. 720291, 1:50 IF, 1:300 Immunoblot), anti-Neurofilament 200 (Sigma Aldrich, cat. N5389, 1:200 IF), anti-β3-tubulin (Synaptic System, cat. 302 302, 1:300 IF), anti-histone H3 (rabbit polyclonal, H3F3A, Abcam, Cambridge, UK, cat. ab1791, 1:2000 Immunoblot). Secondary antibodies conjugated to Alexa Fluor 488 or Alexa Fluor 555 and α-bungarotoxin (α-BTx)-Alexa-555 (cat. B35451, 1:200) were from ThermoFisher. Anti-mouse and anti-rabbit IgG HRP-conjugated were purchased from Calbiochem (Sigma Aldrich). Urocortin 2 peptide was synthetised by Caslo ApS (Kongens Lyngby, Denmark) following the known sequence of the mature murine peptide (VILSLDVPIGLLRILLEQARYKAARNQAATNAQILAHV UniProt #Q99ML). Astressin 2B was purchased from Sigma (cat. A5227). Fluorescent Mounting Medium was purchased from Dako Agilent (Santa Clara, CA, USA). µ-Conotoxin GIIIB (cat.C-270) and α-LTx (cat. LSP-130) were purchased from Alomone (Jerusalem, Israel). The purity of the toxin was checked by SDS–PAGE, and its neurotoxicity by ex vivo mouse nerve-hemidiaphragm preparations as previously described [52].

### 4.2. Animals

C57BL/6 mice expressing cytosolic GFP under the promoter of the *PLP* gene [53], kindly provided by Dr. W.B. Macklin (Aurora, Colorado) with the help of Dr. T. Misgeld (München, Germany), were used in immunofluorescence experiments. CD1 mice weighting around 25 gr were employed for electrophysiological recordings and imaging. Mice were maintained under a 12-h light/12-h dark cycle and constant temperature in the animal facility of the Department. Water and food were available ad libitum. Paralysis was restricted to one hind limb and did not impair food or water intake. Tissue sampling was carried out in animals sacrificed under deep anaesthesia.

### 4.3. Sample Preparation for NMJ Laser Microdissection

Transgenic C57BL/6 female mice of 20–25 g were injected intramuscularly at the level of the outer thigh of the hind limb with α-LTx (5 μg/kg in 15 μL of 0.9% NaCl, 0.2% gelatin) upon isoflurane anesthetisation. Animals were randomised based on their weight, and control animals were injected with vehicle. To localise the NMJs α-BTx Alexa-555 (1:200 dilution in 0.9% NaCl, 0.2% gelatin solution) was locally injected 1 h before sacrifice. At indicated time points, mice were sacrificed according to European guidelines, *Levator auris longus* (LAL) or soleus muscles were dissected and fixed in 4% PFA (wt/vol) for 15 min. Samples were then frozen in liquid nitrogen, cryosliced (10 μm thick), and transferred to UV-treated microscope glass slides. Microdissection was performed under direct microscopic visualisation with PALM RoboMover automatic laser microdissector (Carl Zeiss, Oberkochen, Germany).

### 4.4. RNA Extraction and Bioinformatic Analysis

Total RNA was isolated from the microdissected samples by incubation as previously described [15]. Briefly, tissue was lysed through o/n incubation with 50 μL of lysis buffer PKD (Qiagen, Venlo, Netherlands) and 10 μL of proteinase K solution (Promega, Madison, WI, USA) at 55 °C. After centrifugation for 10 min at 6700 g and RNA was extracted using the Maxwell^®^ 16 LEV RNA FFPE Purification Kit (Promega) following manufacturer’s instructions. To prepare cDNA from RNA samples, the SMARTer Universal Low Input RNA kit (Takara Bio USA, Mountain View, CA, USA) was employed following manufacturer’s instructions. Libraries were prepared using the Ion TargetSeq Exome Enrichment kit, following the manufacturer’s guidelines. Ion PI Sequencing 200 kit (Ion Torrent, ThermoFisher). The Ion PI Chip (Ion Torrent) was prepared and calibrated for loading. The Ion PI Chip was loaded with a template-positive ISPs and run on the Ion Proton Sequencer. Bioinformatics analysis was carried out using several command line software included in Bio-Linux. Using the Star aligner, the reads, previously filtered for quality and length, were processed and aligned to mouse genome (mm10 version). The unmapped reads generated from the first step, were re-aligned by using Bowtie2. The reads mapped with Star and Bowtie2 were merged and processed with Cufflinks. Cufflinks uses the alignment file to assemble and reconstruct the transcriptome. Cuffdiff (included in Cufflinks) was used to calculate the differential gene expression between different groups (0, 4, 24, and 96 h). Cuffdiff calculates expression in two or more samples and tests the statistical significance of each observed change in expression between them. The statistical model used to evaluate changes assumes that the number of reads produced by each transcript is proportional to its abundance but fluctuates because of technical variability during library preparation and sequencing and because of biological variability between replicates of the same experiment. RNA-seq data have been deposited in the ArrayExpress database at EMBL-EBI (www.ebi.ac.uk/arrayexpress) under accession number E-MTAB-5730. The dataset was then analysed with the following workflow. We selected transcripts that showed a significant differential expression compared to the control in at least one time point of the kinetics (indicated as differentially expressed genes -DEGs). Among them, we selected only the up-regulated transcripts. We analysed these hits with the online analysis tool WebGestalt to perform a gene ontology (GO) analysis. For this analysis, we chose low stringency criteria in order to highlight all the macro-categories of the GO classification represented in our list of transcripts. In details, the advanced parameters used include: the minimum number of gene for category = 1, FDR = 1, and the number of categories expected and visualised set to the maximal score possible. We then focused on the macro-category called extracellular proteins and performed an additional screening, using a database, e.g., Uniprot and/or Reactome, by choosing only small peptides, hormone, and growth factors, as we were interested in regeneration-promoting factors. Finally, we included in our search those factors that were strongly upregulated early in the process and then return to control levels.

### 4.5. Droplet Digital PCR

Droplet digital PCR (ddPCR) for *UCN2* was carried out using GAPDH as an internal control following manufacturer’s instructions. Briefly, reactions were performed in triplicate in a 96-well plate using 10 μL/reaction of 2× ddPCR Supermix for Probes (No dUTP), 1 μL/reaction of 20× target primers/probe (FAM or HEX, Bio-Rad, Hercules, CA, USA), 1 μL/reaction of 20× reference primers/probe (FAM or HEX, Bio-Rad), 3 μL cDNA and 5 μL H_2_O. All steps used a ramp rate of 2 °C/s. Results were analysed in the QX200 Droplet Reader, the RNA targets were quantified using the QuantaSoftTM software. Results were expressed as fractional abundance. The primers for the detection were generated using the PrimePCR™ ddPCR™ Expression Probe Assay designed by Bio-Rad: *UCN2*-FAM (ID: dMmuCPE5103910 Bio-Rad) and GAPDH-HEX (ID: dMmuCPE5195283, Bio-Rad).

### 4.6. Neuronal Cultures

Primary rat spinal motoneurons (SCMNs) were isolated from Sprague-Dawley rat embryos (embryonic day 14) and cultured following previously described protocols [54]. Briefly, spinal cords were dissected from E14 rat embryos, treated with trypsin (0.025% m/w) and DNase (0.1 mg/mL) and collected under a bovine serum albumin (BSA) cushion. Cells were then resuspended in Neurobasal medium (Life Technologies) supplemented with 2% B27 supplement (Life Technologies), 2% horse serum (Euroclone), 0.5 mM glutamine, 25 μM 2-mercaptoethanol, 10 ng/mL CNTF (R&D Systems, Minneapolis, MN, USA), 100 pg/mL GDNF (R&D Systems), 5 μg/mL Pen/Strep and 25 μM L-glutamic acid, and seeded on poly ornithine and laminin coated plates. Cultures were maintained at 37 °C in a humidified atmosphere of 95% air, 5% CO_2_.

Rat primary cerebellar granular neurons (CGNs) were prepared from 6-day-old Wistar rats as previously described [28]. Briefly, neurons were isolated from freshly dissected cerebella by mechanical disruption in the presence of trypsin (0.08% m/w) and DNase I (0.08 mg/mL) and then seeded onto 24-well culture plates coated with poly L-lysine (10 μg/mL). Cells were seeded at a density of 3 × 10^5^/well in BME (Life Technologies) supplemented with 10% FBS (Euroclone, Milan, Italy), 25 mM KCl, 2 mM glutamine and 50 μg/mL gentamycin. Cultures were maintained at 37 °C in a humidified atmosphere of 95% air, 5% CO2. Cytosine arabinoside (10 μM) was added to the culture medium 18–24 h after plating to arrest the growth of non-neuronal cells. Experiments were performed at 6 days in vitro.

To test the effect of UCN2 on axon growth, low-density SCMNs cultures were used immediately after plating and exposed for 24 h to UCN2 100 nM, or to a combination of A2B and UCN2 (10 μM and 100 nM respectively) in culture medium. Immunofluorescence was performed following fixation with a 4% PFA solution.

To monitor CRHR2 expression, cells were fixed at different time points after plating (i.e., 24 h and 7 days) in 4% PFA (wt/vol) in PBS, quenched 10 min in 50 nM NH_4_Cl, and permeabilised for 3 min at RT with 0.3% Triton X-100 in PBS. After saturation with 3% Goat serum in PBS for 1 h, samples were incubated with primary antibody against CRHR2 and Neurofilament in 3% goat serum in PBS overnight at 4 °C. After washing and incubation with corresponding Alexa-conjugated secondary antibodies for 1 h at RT, coverslips were mounted using Fluorescent Mounting Medium. Axon length was evaluated by epifluorescence (Leica DMIRE2, Leica Microsystems, Wetzlar, Germany), and axonal length quantified using the ImageJ plugin NeuronJ as described in [18,19].

### 4.7. Electrophysiological Recordings of Evoked EndPlate Potentials (EPPs)

To test the ability of UCN2 to promote functional recovery, mice were injected in the hind limb with α-LTx (5 μg/kg in 15 μL of 0.9% NaCl, 0.2% gelatin) w/o daily i.m. injections of 200 μg/Kg UCN2 (dissolved in 20 μL of 0.9% NaCl, 0.2% gelatin), or vehicle, and soleus muscles were collected 72 h later. For experiments with the CRHR2 antagonist Astressin 2B, mice were i.m. injected daily with 200 μg/Kg A2B (in 20 μL of 0.9% NaCl, 0.2% gelatin), or vehicle, after local injection of α-LTx (as described above), and soleus muscles were dissected out 96 h later. After dissection soleus muscles were immediately placed in an experimental chamber for recording and bathed with an oxygenated (95% O_2_ and 5% CO_2_) Krebs-Ringer solution at RT (20–22 °C). Intracellular recordings of EPPs were performed using intracellular glass microelectrodes (20 MΩ resistance, Science Products, Germany) filled with a 1:2 solution of 3 M KCl and 3 M CH_3_COOK as previously described [55]. Evoked neurotransmitter release was recorded in current-clamp mode, and resting membrane potential was adjusted with current injection to −70 mV. EJPs were elicited by supramaximal soleus nerve stimulation at 0.5 Hz using a suction microelectrode (Science Products, Germany) connected to a S88 stimulator (Grass, West Warwick, Rhode Island, USA) through a stimulus isolation unit (SIU5, Grass). To prevent muscle contraction, excised muscles were incubated for 10 min with 1 μM μ-Conotoxin GIIIB (Alomone Lab, Jerusalem, Israel). Signals were amplified with an intracellular amplifier (BA-01X, NPI, Tamm, Germany), sampled at 10 kHz using a digital interface (NI PCI-6221, National Instruments, Austin, TX, USA) and fed to a PC for recording using an appropriate software (WinEDR, Strathclyde University, Glasgow, UK). EPPs peak amplitude was measured offline with pClamp9 (Molecular Devices LLC, San Jose, CA, USA).

### 4.8. NMJ Immunofluorescence

After electrophysiological recording, soleus muscles were immediately fixed in 4% PFA (wt/vol) in PBS for 10 min at RT. After quenching for 20 min in 50 mM NH_4_Cl in PBS, muscles were separated in bundles of 10–20 myofibres under a dissection microscope as previously described [56]. After saturation for 2 h in blocking solution (15% goat serum, 2% BSA, 0.25% gelatine, 0.20% glycine, 0.5% Triton X-100 in PBS), samples were incubated with anti-VAMP1 primary antibody in blocking solution for 72 h at 4 °C. Bundles were then washed with PBS, and incubated with Alexa Fluor 488 secondary antibody and α-BTx Alexa-555 in PBS for nAChR. Structural recovery was assessed by counting the number of junctions positive both for VAMP1 and α-BTx. Images were collected with a Leica SP5 Confocal microscope (Leica Microsystems, Wetzlar, Germany), equipped with a 40× HCX PL APO NA 1.4 oil immersion objective. Laser excitation line, power intensity and emission range were chosen accordingly to each fluorophore in different samples to minimise bleed-through.

### 4.9. Microfluidic Devices

Microfluidic chambers were produced using established methods [57]. Polydimethylsiloxane (Dow Corning, Midland, MI, USA) inserts were sterilised and fixed to 50 mm glass-bottomed dishes (WillCo Wells B.V., Amsterdam, Netherlands) using plasma cleaning. The chambers were blocked with 0.8% BSA in PBS overnight at 37 °C and then coated with poly L-lysine. SCMNs were plated in the somatic compartment and observed until axons reached the distal chamber, typically within 6–7 days in vitro, when neurons were fixed and stained with the indicated antibodies as describe before [15].

### 4.10. Western Blotting

Cells were lysed in Lysis Buffer (Hepes 10 mM, NaCl 150 mM, SDS 1%, EDTA 4 mM, protease and phosphatase inhibitors). Protein concentration was quantified using BCA assay (Protein Assay Kit, Thermo Fisher). Ten micrograms of total cell lysates were loaded into NuPage 4–12% Bis-Tris gels and separated by electrophoresis in MES buffer (Life Technologies, B0002). For Western blotting, proteins were transferred onto Protran nitrocellulose membranes and saturated for 1 h in PBS-T (PBS, 0.1% Tween 20) supplemented with 5% BSA (Sigma, A4503-100G). Incubation with primary antibodies was performed o.n. at 4 °C. Membranes were washed and incubated with specific HRP-conjugated secondary antibodies for 1 h. After additional washings, signals were revealed with Luminata^TM^ using an Uvitec gel doc system (Uvitec, Cambridge, UK).

### 4.11. Statistical Analysis

Sample sizes were determined by analysis based on data collected by our laboratory in published studies. We used at least *n* = 4 mice/group for electrophysiological analysis, *n* = 11 fibres recorded. We ensured blinded conduct of experiments. For cell cultures studies, at least 3 independent replicates were performed. Axon length quantification was conducted by an observer who was blind to the experimental groups. No samples or animals were excluded from the analysis. Data displayed as histograms are expressed as means ± SEM or SD. GraphPad Prism software was used for all statistical analyses. Statistical significance was evaluated using analysis of variance (ANOVA) with Tukey post-test or two-tailed, unpaired Student’s *t*-test depending on group number. Data were considered statistically different when * *p* < 0.05, ** *p* < 0.01, *** *p* < 0.001, **** *p* < 0.0001.

## Data Availability

The datasets generated for this study have been deposited in the repository of the EMBL’s European Bioinformatics Institute (EMBL-EBI) at the following link: https://www.ebi.ac.uk/arrayexpress/experiments/E-MTAB-5730/files/ (access date: 1 December 2021).
